# Tomato lipidic extract plus selenium decrease prostatic hyperplasia, dihydrotestosterone and androgen receptor expression versus finasteride in rats

**DOI:** 10.1007/s00345-023-04558-x

**Published:** 2023-09-02

**Authors:** David Julian Arias-Chávez, Patrick Mailloux-Salinas, Jessica Ledesma-Aparicio, Elihu Campos-Pérez, Omar Noel Medina-Campos, José Pedraza-Chaverri, Guadalupe Bravo

**Affiliations:** 1 Departamento de Farmacobiología, Centro de Investigación y de Estudios Avanzados del IPN, Sede Sur, Ciudad de México, Mexico; 2 Departamento de Patología, Hospital General Dra Matilde Petra Montoya Lafragua, ISSSTE, Ciudad de México, Mexico; 3 Departamento de Patología, Hospital Ángeles Lindavista, Ciudad de México, Mexico; 4 Laboratorio F-315, Departamento de Biología, Facultad de Química, Universidad Nacional Autónoma de México, Ciudad de México, Mexico

**Keywords:** Prostatic hyperplasia, Dihydrotestosterone, Tomato, Selenium, Finasteride, Androgen receptor

## Abstract

**Purpose:**

Evaluate the therapeutic effect of a tomato lipidic extract (STE) in combination with selenium (Se) on rats with prostatic hyperplasia (PH) and to observe its possible mechanisms of action and synergism versus finasteride.

**Materials and methods:**

54 male Wistar rats of nine weeks old were divided in Control (C), PH, Finasteride (F), STE, Se, F + STE, F + Se, STE + Se and F + STE + Se with testosterone enanthate (except C). After 4 weeks of treatment administration, prostate weight, bladder weight, diuresis, prooxidant and antioxidant activity, dihydrotestosterone (DHT), androgen receptor (AR) expression and anatomopathological analysis were determined.

**Results:**

STE + Se decreased prostate weight 53.8% versus 28% in F group, also STE + Se decreased significatively glandular hyperplasia, prooxidant activity, DHT and AR expression and increased diuresis and antioxidant activity versus finasteride which increased MDA in prostate.

**Conclusions:**

These results demonstrate a greater therapeutic and beneficial effect of tomato lipidic extract in combination with Se in young rats with PH with respect to finasteride without increase prooxidant activity.

**Supplementary Information:**

The online version contains supplementary material available at 10.1007/s00345-023-04558-x.

## Introduction

Benign prostatic hyperplasia (BPH) is the most the most frequent benign tumor in the men [1, 2]. The increase in prostate volume causes bladder obstruction and physical compression of the urethra leading to discomfort clinically referred to as lower urinary tract symptoms (LUTS) [1, 2]. The molecular mechanisms leading to BPH remain unclear; however, dihydrotestosterone (DHT) is known to be strongly involved in the pathophysiology of BPH [3–5]. DHT has a higher affinity for the androgen receptor (AR) than testosterone, making it an acute mediator of BPH. DHT-AR complex regulates the expression of target genes, resulting in different biological responses such as differentiation, proliferation, and survival [4, 5].

The current first-line pharmacological treatments for BPH are alpha-blockers and 5-apha reductase inhibitors (5-ARIs) [6]. The 5-ARIs, such as finasteride, generates different adverse effects like, erectile dysfunction and others [6, 7]. Phytotherapeutic treatment has been postulated as a therapeutic alternative for BPH [1, 8]. Particularly, tomato, has been widely reported as a rich source of bioactive compounds such as minerals, vitamin, tetraterpenes and polyphenols [8–11]. These compounds have been individually reported to have anti-inflammatory, antioxidant, antiproliferative and proapoptotic activity, some of them also induce a down-regulation of the enzyme 5-α-reductase and of AR, therefore, they reduce DHT levels and decrease the size of the prostate [8–11].

Selenium (Se) generates selenoproteins of great biological importance in health such as glutathione (GSH), glutathione peroxidase (GPx) and thioredoxin reductases that promote an optimal antioxidant/oxidant balance, pro-apoptotic activity, as well as anti-inflammatory and antiproliferative effects [12–14]. It has been described that extract of *Serenoa repens* plus lycopene and Se alone or in combination had greater therapeutic efficacy than individually in reducing prostate size, proinflammatory markers, growth factors, and pro-oxidants such as malondialdehyde (MDA) and nitrites (–NO_2_) in preclinical and clinical protocols for BPH [1, 14, 15]. However, there are no reports of the possible beneficial effect of a lipidic extract of whole tomato in combination with Se for the treatment of BPH. The aim of this work is to evaluate the therapeutic effect of a lipid extract of whole tomato in combination with Se in rats with prostatic hyperplasia (PH) compared to finasteride.

## Materials and methods

### Experimental animals

Fifty-four male Wistar rats of 9 weeks old were obtained from Cinvestav-IPN Pharmacobiology Department’s animal facility. Animals were housed in an environmentally controlled room with a 12-h light–dark cycle. All animals received LabDiet 5008^®^ rat chow (Richmond, IN, USA) and water ad libitum. The animals were randomized into nine groups (n = 6 rats/group); control (C), PH, finasteride (F), tomato lipidic extract (STE), selenium (Se), F + STE, F + Se, STE + Se and F + STE + Se. Except for the control group, which received corn oil vehicle s.c., all animals were injected with testosterone enanthate (Testoprim-D^®^) 10 mg/kg three times a week for 4 weeks s.c. Testosterone induction was maintained until the end of the study. The doses used were finasteride (TEALEP^®^): 5 mg/kg/day; lipidic extract of whole tomato (Mexican patent No. 380295): 5 mg/kg/day (with respect to lycopene concentration); selenium (Sigma-Aldrich^®^): 10 μg/kg/day. BPH group received corn oil as vehicle. The reagents are described in the supplementary material.

### Diuresis

On the last day of treatment, the animals were placed in acrylic metabolic boxes with water ad libitum to collect urine output for 12 h. The volume of water consumed, and the urinary volume were quantified.

### Prooxidant activity

MDA and nitrites (–NO_2_) assays were performed in the homogenized prostate as previously reported [1]. Antioxidant enzyme assays are described in the supplemental material.

### Dihydrotestosterone assay

DHT was determined by enzyme-linked immunosorbent assay (ELISA, FineTest^®^ Catalogue:EU2551) according to the manufacturer’s instructions. Samples were placed in a sensitized microplate and incubated. Optical density was read at 450 nm.

### Androgen receptor expression

50 mg of macerated tissue was mixed with 1 mL of RIPA buffer supplemented with a cocktail of phosphatase and protease inhibitors (2 µg/mL aprotinin, 2 µg/mL leupeptin, 1 µg/mL pepstatin, 0.1 mM PMSF, 50 mM NaF and, 1 mM Na_3_VO_4_). Proteins were separated with 14% SDS-PAGE gels, transferred to polyvinylidene difuoride membranes, and then incubated with primary antibodies at the indicated dilution; 1:1000 for AR (Abcam) and 1:1500 for GAPDH (Santa Cruz Biotechnology). After were incubated with anti-rabbit 1:1000 for 2 h at room temperature. 1 ml of Western HRP Substrate for chemiluminescent detection was added. Chemiluminescent proteins were revealed in a dark room using Kodak X-ray Plates, and bands intensity quantification was performed by densitometry using Image Studio Lite V. 5.2 (Li-Cor).

### Anatomopathological analysis

Prostates were fixed in 10% formalin, dehydrated, and fixed in paraffin. The tissues were cut in 7-μm slices, stained with hematoxylin–eosin. The analysis was carried out in detail by the anatomopathologist. Morphometric measurements of the prostate gland were analyzed using FIJI ImageJ 1.53j.

### Statistical analyses

All the data were analyzed using GraphPad Prism 9.0 software. Data are presented as mean ± SEM. The experimental groups were compared using analysis of variance (ANOVA) and a post hoc Tukey test. Values p ≤ 0.05 considered significant.

## Results

### Body and prostate weight

It was observed a significant decrease in body weight in the PH group compared to the C group (Table 1). All the groups that received treatments did not present significant differences with respect to C. The prostate weight of the PH group presented a significant difference with respect to the C group with an increase 2.58-fold due to the administration of testosterone. The F group had a decrease of 28% with respect to the PH group, while the STE and Se groups had a decrease of 47.3% and 45.7% compared to PH, respectively. The same trend was observed with the F + STE and F + Se groups (decrease of 45.2% and 41.9% with PH, respectively). The STE + Se and F + STE + Se groups had the greatest decrease in prostate gland weight, with a 53.8% and 53.2% decrease with respect to PH and F, but no difference with C after 4 weeks of treatment (Table 1). On the other hand, F group showed the lowest % inhibition of prostatic growth (45.6%) with respect to STE and Se (77.2% and 74.6%), but no differences when F was administered in combination. STE + Se and F + STE + Se were the groups that had the highest inhibition (87.6% and 86.8%, respectively) of prostate gland growth. Finally, the bladder of the PH group with respect to C was significantly larger. In comparison with PH, F group decreased bladder weight, but it was significantly higher with respect to STE and Se alone or in combination with F. Otherwise, STE + Se and F + STE + Se had a significant decrease, as well as STE and Se, had no differences with group C.

**Table 1:** Comparisons between groups for the different parameters assessed

	C	PH^t^	F^t^	STE^t^	Se^t^	F + STE^t^	F + Se^t^	STE + Se^t^	F + STE + Se^t^
Body weight (g)	518.8 ± 19.07	400.8 ± 22.52*	453 ± 17.58	466.5 ± 16.09	431.8 ± 33.06	444.7 ± 18.89*	442.8 ± 12.79*	480.2 ± 8.71	468.7 ± 4.96
Prostate weight (g)	0.72 ± 0.374	1.86 ± 0.041*	1.34 ± 0.046*^,a^	0.98 ± 0.017*^,a,b^	1.01 ± 0.032*^,a,b^	1.02 ± 0.061*^,a,b^	1.08 ± 0.031*^,a,b^	0.86 ± 0.022^a,b,c^	0.87 ± 0.033^a,b,c^
Prostatosomatic index (%)	0.14 ± 0.004	0.47 ± 0.034*	0.29 ± 0.010*^,a^	0.21 ± 0.009*^,a,b^	0.24 ± 0.026*^,a,b^	0.23 ± 0.008*^,a,b^	0.25 ± 0.011*^,a,b^	0.18 ± 0.004^a,b.c^	0.19 ± 0.007^a,b,c^
Decrease in PW (%)	–	–	28	47.3	45.7	45.2	41.9	53.8	53.2
Growth inhibition (%)	–	–	45.6	77.2	74.6	73.7	68.4	87.7	86.8
Bladder weight (g)	0.11 ± 0.004	0.22 ± 0.004*	0.19 ± 0.010*^,a^	0.12 ± 0.005^a,b^	0.14 ± 0.007*,^a,b^	0.12 ± 0.004^a,b^	0.13 ± 0.006^a,b^	0.11 ± 0.003^a,b^	0.11 ± 0.004^a,b^
Bladder weight/Body weight (%)	0.021 ± 0.001	0.057 ± 0.004*	0.042 ± 0.002*^,a^	0.027 ± 0.002^a,b^	0.033 ± 0.004^a,b^	0.028 ± 0.002^a,b^	0.030 ± 0.002^a,b^	0.023 ± 0.001^a,b^	0.023 ± 0.002^a,b^
Water (mL/12 h)	42.33 ± 4.60	17.83 ± 1.99*	26.33 ± 4.30*	27.67 ± 5.80*^,a^	24.33 ± 4.96*	28.67 ± 3.82*	20.08 ± 4.81*	44.67 ± 1.35^a,d,f^	41.33 ± 1.43^a,d^
Diuresis (mL/12 h)	31.83 ± 2.89	6.833 ± 0.65*	13.5 ± 0.76*	21.17 ± 2.44*^,a^	18.83 ± 2.91*^,a^	19.05 ± 2.42*^,a^	18.5 ± 2.20*^,a^	29.33 ± 1.64^a,b,d,e,f^	28.5 ± 1.29 ^a,b,d,e,f^
MDA (mmol/mg)	1.09 ± 0.06	3.33 ± 0.36*	4.74 ± 0.64*^,a^	1.43 ± 0.10*^,a,b,g^	1.69 ± 0.08*^,a,b,g^	1.69 ± 0.09*^,a,b,g^	1.98 ± 0.20*^,a,b.c.g^	1.05 ± 0.11^a,b^	1.78 ± 0.05*^,a,b,g^
–NO_2_ (μmol/mg)	15.78 ± 0.78	33.5 ± 1.54*	37.82 ± 1.75*^,a^	21.68 ± 0.61*^,a,b^	24.33 ± 0.76*^,a,b^	26.08 ± 1.51*^,a,b^	29.88 ± 1.56*^,a,b,c^	16.86 ± 1.06 *^,a,b,c,d,e,f^	19.49 ± 0.81*^,a,b,d,e,f^

*C* control, *PH* prostatic hyperplasia, *F* finasteride, *STE* tomato lipidic extract, *Se* selenium, *PW* prostate weight, *MDA* malondialdehyde, *–NO*_*2*_ nitrites. Prostatosomatic index % was determined for relation between prostate weight and body weight × 100. Values represented as mean ± s.e.m. ANOVA one way. Post hoc Tukey. *p < 0.05 vs C. ^a^p < 0.05 vs PH. ^b^p < 0.05 vs F. ^c^p < 0.05 vs STE. ^d^p < 0.05 vs Se. ^e^p < 0.05 vs F + STE. ^f^p < 0.05 vs F + Se. ^g^p < 0.05 vs STE + Se

### Diuresis

The consumption of water was significantly lower in PH with respect to C. F, STE and Se groups and their combinations showed a lower effect versus than that of PH, but only STE + Se and F + STE + Se did not present differences compared to C (Table 1). Diuresis in PH was significantly lower compared to C. F group exhibits no difference with PH, while STE, Se, F + STE and, F + Se showed a significant decrease versus C, but it was significantly higher compared to PH. STE + Se and F + STE + Se being the groups that were not differently significant versus C (Table 1).

### Prooxidant activity

MDA and –NO_2_ levels increased significantly in PH compared with C, while F group had the highest level of MDA and –NO_2_ compared to PH. STE and Se groups had a significant decrease in both prooxidant markers with respect to PH. This same trend was observed with the groups F + STE, F + Se and F + STE + Se, while STE + Se group was not different from C. (Table 1). Antioxidant enzyme levels are reported in Supplementary Fig. 1.

### Dihydrotestosterone levels

**Fig. 1:**
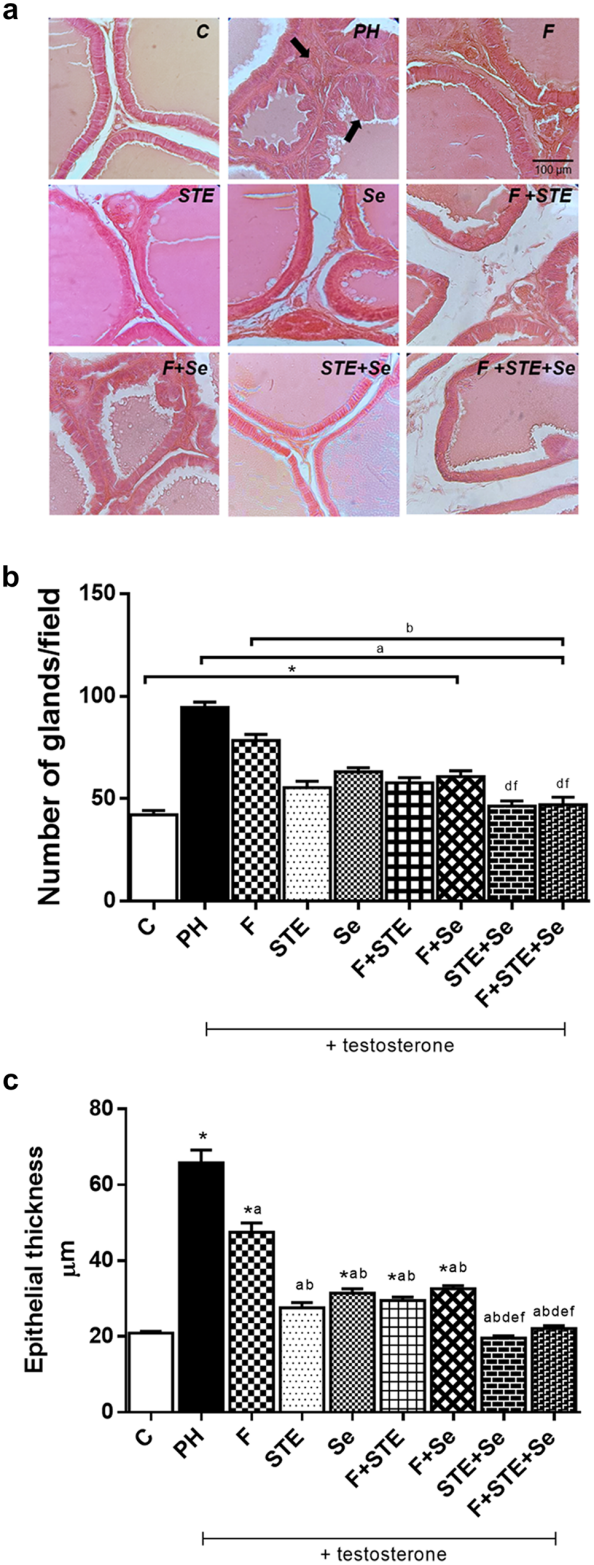
Anatomopathological analysis. **A** Staining of ventral prostate with Hematoxylin–Eosin. View 40×. **B** Number of glands/field. **C** Epithelial thickness. C, control; PH, prostatic hyperplasia; F, finasteride; STE, *Solanum* *lycopersicum* fruit lipidic extract; Se, selenium. Values represented as mean ± s.e.m. ANOVA one way. Post hoc Tukey. Values represented as mean ± s.e.m. ANOVA one way. Post hoc Tukey. *p < 0.05 vs C. ^a^p < 0.05 vs PH. ^b^p < 0.05 vs F. ^d^p < 0.05 vs Se. ^f^p < 0.05 vs F + Se. n = 6. ↓: Proliferative epithelium. Scale bars: 100 μm

The DHT levels in PH group, significantly increased versus C. All groups presented a significant decrease with respect to PH, except for the Se group, in both serum and prostatic tissue without being different with C (Fig. 1A, B). This suggest that STE has the same effect as F.

### Androgen receptor expression

AR expression was significantly increased in the PH, and F group compared to C. The F group decreases compared to PH, but STE and Se alone or in combination with F show significant differences compared to PH and the F group, while those STE + Se and F + STE + Se groups did not differ from C. (Fig. 1C). This suggests that STE and Se decreases cell proliferation by decreasing AR.

### Anatomopathological analysis

**Fig. 2:**
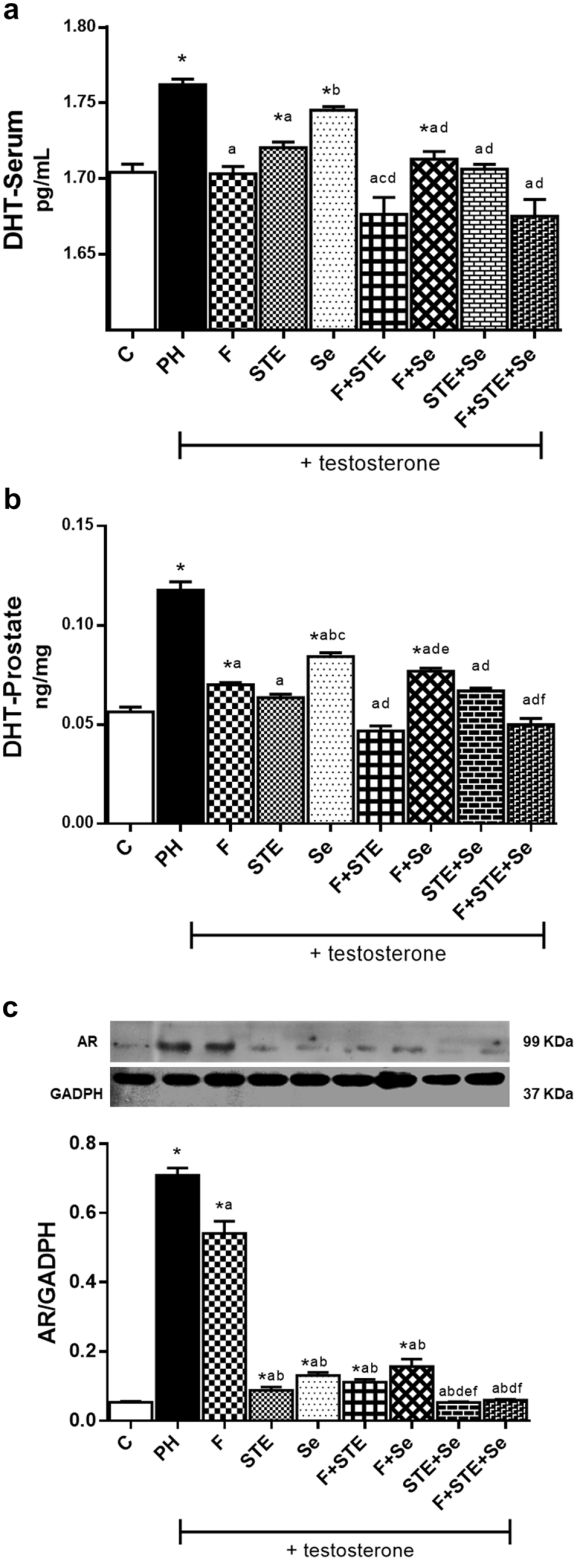
DHT levels and Androgen receptor expression. **A** DHT in serum. **B** DHT in prostate. **C** Inmunoblots and Western Blot analysis of AR expression. C, control; PH, prostatic hyperplasia; F, finasteride; STE, *Solanum lycopersicum* fruit lipidic extract; Se, Selenium; AR, androgen receptor; DHT, Dihydrotestosterone. Values represented as mean ± s.e.m. ANOVA one way. Post hoc Tukey. *p < 0.05 vs C. ^a^p < 0.05 vs PH. ^b^p < 0.05 vs F. ^c^p < 0.05 vs STE. ^d^p < 0.05 vs Se. ^e^p < 0.05 vs F + STE. ^f^p < 0.05 vs F + Se. ^g^p < 0.05 vs STE + Se. n = 6

The C group presented a microarchitecture of a normal prostate. (Fig. 2A–C). The PH group showed glandular hyperplasia characterized by an increase in the number of glands, loss of stroma and a significant increase in the thickness of the glandular epithelium compared to C. F group showed a significant decrease in the number of glands and in the epithelial thickness. On the other hand, STE and F + STE groups did not show differences between them. The Se and F + Se groups also did not show differences between them but with group C. Finally, STE + Se and F + STE + Se showed no significant differences between them and group C (Fig. 2A–C).

## Discussion

It has been established that the origin of BPH in men is mediated by androgen stimulation [2, 16]. Exogenous administration of testosterone in experimental animal models has been shown to significantly increase prostate gland weight and DHT [2, 11, 17]. Tomato is a rich source of vitamins (A, B, C, D and E), minerals (Zn, Mn, Cu, Fe), Carotenoids (lycopene, α and β-carotene), and polyphenols (quercetin, myricetin, kaempferol, daidzein, and other), present in the skin, pulp and seeds, which have been reported to decrease prostate size both in vitro and in vivo, as well as decreasing LUTS and increasing flow in urine [9–11]. According to these results, the administration of STE compared to finasteride had a greater effect in reversing hyperplasia. Compared to the combination with Se, the effect was greater. These results agree with previous reports, where the combination of pure lycopene, Se, and *S. repens* had a greater effect in decreasing prostate gland weight [14, 15]. However, adverse effects have been reported for the use of *S. repens* like those reported for finasteride such as erectile dysfunction [18].

Selenium has been reported to possess antiproliferative, anti-inflammatory and pro-apoptotic effects for which it has been used as an adjuvant in the treatment of BPH [14, 19]. According to our results, although separately the extract and Se significantly decreased prostate weight with respect to the PH group, the STE + Se combination had the ability to completely reverse the hyperplasia in only 4 weeks of treatment, despite the administration of testosterone for 8 weeks. The decrease in prostatic hyperplasia was directly confirmed by anatomopathological analysis showing that STE + Se was more effective than finasteride. Finasteride aims to inhibit the conversion of testosterone to DHT preventing prostatic cell proliferation; however, this effect is a long time (2 to 6 months) [6, 20]. This could explain why the administration of finasteride was not sufficient to reverse glandular hyperplasia and increase diuresis in 4 weeks. Because of this, it is usually administered in combination with an alpha-adrenergic blocker to decrease LUTS so that finasteride can have the desired therapeutic effect [6].

Patients with BPH, in addition to urinary tract infections due to prolonged concentration of urine in the bladder, as an adaptive response to urine volume, the bladder may present morphological changes to compensate for greater volume by increasing its volume, generating bladder hyperactivity due to prostatic enlargement [21, 22]. In our results, the groups that presented greater prostate and bladder weight probably due to greater narrowing and less urine emptying due to enlargement prostatic. On the other hand, it was also observed that, unlike finasteride, STE and Se increased diuresis probably due to a decrease in bladder obstruction. This suggests that monotherapies and their combination (STE + Se) can reduce LUTS in less time compared to finasteride.

The accumulated production of ROS due to endogenous or exogenous causes plays a determinant role in diseases such as BPH [2, 23]. Furthermore, finasteride with one month of treatment, despite reducing DTH levels, was the treatment that induced the least reduction in prostate size and increased MDA and -NO_2_ levels. This is related to one of its main adverse effects which is erectile dysfunction because free radicals rapidly sequester the nitric oxide responsible for vasodilation so that erection takes place [7]. Adverse effects reported from the use of finasteride during and after treatment for long time, known as post-finasteride syndrome, in addition to erectile dysfunction, are gynecomastia, loss or reduction of libido, ejaculatory dysfunction, insomnia and psychiatric illnesses [6, 7].

The inhibition of 5-α-reductase, reduction of prostate-specific antigen and DHT, inhibition of IGF-1 signal transduction and inhibition of androgen-mediated signaling by down-regulation of AR have been reported for administration of polyphenols and other bioactive compounds presents in tomato [6, 9, 11, 23–25]. This could explain why the groups receiving STE alone or in combination had a decrease DHT levels and AR expression. However, together with Se, the therapeutic effects are better in reducing these markers of BPH compared to finasteride. The decrease of AR expression in the Se group could be explained by its antiproliferative and pro-apoptotic effects, thus by decreasing the number of cells, including epithelial cells where AR is commonly expressed [5]. However, further studies are needed to know whether the results obtained in rat prostates can be reproduced in men with BPH to know the possible mechanism of action responsible for this effect.

## Conclusions

Our results demonstrate that STE plus Se compared to finasteride decrease the alterations generated by PH in this animal model. This work would be a proposal as a safe and effective therapeutic alternative for the treatment of BPH in men without the adverse effects produced by the 5-ARIs.

### Supplementary Information

Below is the link to the electronic supplementary material.

Supplementary file1 (DOCX 116 KB)

## Data Availability

The datasets generated in the current study are available from the corresponding author on reasonable request.
