# Supplementary material for: Tomato lipidic extract plus selenium decrease prostatic hyperplasia, dihydrotestosterone and androgen receptor expression versus finasteride in rats
Source: World J Urol. 2023 Sep 2;41(10):2793–9. doi: 10.1007/s00345-023-04558-x (PMC10582118; doi:10.1007/s00345-023-04558-x)
Supplement: Supplementary file 1 — Supplementary file1 (DOCX 116 KB) [file 345_2023_4558_MOESM1_ESM.docx]

**Tomato lipidic extract plus selenium decrease prostatic hyperplasia, dihydrotestosterone and androgen receptor expression versus finasteride in rats**

David Julian Arias-Chávez^1^, Patrick Mailloux-Salinas^1^, Jessica Ledesma-Aparicio^1^ Elihu Campos-Pérez^2,3^, Omar Noel Medina-Campos^4^, José Pedraza-Chaverri^4^, Guadalupe Bravo^1^

^1^Departamento de Farmacobiología, Centro de Investigación y de Estudios Avanzados del IPN, Sede Sur. México

^2^Departamento de Patología, Hospital General Dra Matilde Petra Montoya Lafragua, ISSSTE. México

^3^Departamento de Patología, Hospital Ángeles Lindavista, México

^4^Laboratorio F-315, Departamento de Biología, Facultad de Química, Universidad Nacional Autónoma de México. México.

Author to whom correspondence should be addressed: Guadalupe Bravo, PhD (E-mail: [gbravof@yahoo.com](mailto:gbravof@yahoo.com)) Department of Pharmacobiology, Cinvestav-IPN, Calz. de los Tenorios 235, Col. Granjas Coapa, 14330 Mexico City., Mexico. FAX. (+52 55) 4832863.

**Supplementary material and methods**

***Reagents***

Finasteride (TEALEP®) was purchased from Asofarma de México (Santa Fe, CDMX, Mexico). Testosterone (Testoprim-D®) was obtained from Tocogino Laboratorios (Cuauhtémoc, CDMX, Mexico). Acetic acid, trichloroacetic acid, thiobarbituric acid, NaF, NaCl and C_24_H_39_NaO_4_ were acquired from J.T. Baker™ (Xalostoc, Edo, Mex. Mexico). Aprotinin, leupeptin, pepstatin, selenium, n-butanol, pyridine, xanthine, xanthine oxidase, ammonium molybdate, potassium phosphate, EDTA, NaN_3_, NADPH, Na₂CO₃, Na_3_VO_4_, HEPES, NP-40, PMSF, SDS, NBT, H_2_O_2_, GR, GSH, were purchased from Sigma-Aldrich (St. Louis, MO. USA). Androgen receptor primary antibody was obtained from Abcam (Cambridge, MA, USA), anti-rabbit secondary antibody from GeneTex™ (Irvine,CA, USA) and bovine serum albumin and GAPDH primary antibody from Santa Cruz Biotechnology (Dallas, TX, USA). Western HRP substrate was purchased from Immobilon-Millipore™ (Darmstadt, Germany). pH 7 PBS was prepared and stored at 4°C for a maximum of 15 days. From the stock solutions, standard solutions to build the calibration curves were daily prepared.

***Samples preparation.*** Once the prostates were removed, they were placed in a CoorsTek™ porcelain mortar with 50 ml of liquid nitrogen to be mechanically pulverized with the pistil. Subsequently, prostate homogenates were obtained by taking a 0.1-gram prostate tissue homogenate to which 1 ml of PBS was added in 1.5 ml Eppendorf tubes. The supernatant was centrifuged at 3000 rpm for 10 minutes and the supernatant was separated into aliquots and stored in Eppendorf tubes at -70°C for later use. On the other hand, 10 ml of truncal blood was placed in PIREX® test tubes; it was centrifuged at 10,000 rpm for 10 minutes and the serum was separated in Eppendorf aliquots at -70ºC for later use.

***Glutathione peroxidase activity***

0.035 ml of sample was added to 0.28 ml of mixture reaction (1 mM EDTA, 1 mM NaN_3_, 0.2 mM NADPH, 1 U/ml of the GR and 1 mM GSH in 50 mM potassium phosphate pH 7.0) and 0.035 ml 2.5 mM H_2_O_2_. Optical density at 340 nm was recorded for 3 min and the activity was calculated from the slope of these lines using the extinction coefficient of NADPH at 340 nm (6.22 l mmol^-1^ cm^-1^). Data were expressed as units (μmoles NADPH oxidized per minute)/mg.

***Superoxide dismutase assay***

20 μl of samples were added to 0.17 ml of the mixture reaction (0.122 mM EDTA, 30.6 μM NBT, 0.122 mM xanthine, 0.006% bovine serum albumin, and 49 mM Na₂CO₃) and then 20 μl 0.1 U/ml xanthine oxidase were added and incubated in a water bath at 27ºC for 15 min and the optical density was read at 560 nm. The amount of protein that inhibited NBT reduction to 50% of maximum was defined as one unit (U) of SOD activity. Results were expressed as U/mg.

***Catalase activity***

7 μl of samples were mixed with 70 μl of 20 mM H_2_O_2_ for 3 min and after 0.25 mL of 16.2 mM ammonium molybdate were added and the optical density at 374 nm was recorded. Data were expressed as KU (micromoles H_2_O_2_ decomposed per minute)/mg.

**Supplementary results**

***Antioxidant activity***

Regarding antioxidant activity, GPx activity, PH was not different from C group. Only the Se, F+Se, STE+Se and F+STE+Se groups increased significantly with respect to PH. (Fig. 1A). For SOD levels, PH group increased with respect to C. On the other hand, STE and Se alone or combined with F were also not different compared to PH, however only STE+Se and F+STE+Se significantly increased with respect to PH. (Fig. 2B). Finally, in CAT enzyme, PH group was significantly lower than C, while F was not different from PH, only STE, STE+Se and F+STE+Se increased compared to PH. (Fig. 2C). These results indicate that the oxidative damage generated by finasteride is greater than testosterone. This suggests that STE and Se in combination avoid this adverse effect generated by finasteride.

***Supplementary Fig 1*** Antioxidant enzymes in prostatic tissue. (A): GPx, (B): SOD, (C): CAT. C: Control. PH: Prostatic Hyperplasia. F: Finasteride. STE: Tomato Lipidic Extract. Se: Selenium. GPx: Glutathione Peroxidase. SOD: Superoxide Dismutase. CAT: Catalase. Values represented as mean ± s.e.m. ANOVA one way. Post hoc Tukey. *p < 0.05 vs C. ^a^p < 0.05 vs PH. ^b^p < 0.05 vs F. ^c^p < 0.05 vs STE. ^d^p < 0.05 vs Se. ^e^p < 0.05 vs F+STE. ^f^p < 0.05 vs F+Se. n=6.

**A**

**B**

**C**
